# BPTF is essential for vaccine-induced germinal center B cell responses

**DOI:** 10.1093/jimmun/vkag126

**Published:** 2026-06-20

**Authors:** Alexandria J Sturtz, Birk K Evavold, Zarifeh Heidari Rarani, Wafaa B Alsoussi, Julian Q Zhou, Hanover C Matz, Hiromi Muramatsu, Wren Simkins, Sameer Kumar Malladi, Katherine M McIntire, Jishnu Das, Jackson S Turner, Norbert Pardi, Ali H Ellebedy

**Affiliations:** Department of Pathology and Immunology, Washington University School of Medicine in St. Louis, St. Louis, MO, United States; Department of Pathology and Immunology, Washington University School of Medicine in St. Louis, St. Louis, MO, United States; Center for Systems Immunology, Department of Immunology, University of Pittsburgh, Pittsburgh, PA, United States; Department of Pathology and Immunology, Washington University School of Medicine in St. Louis, St. Louis, MO, United States; Department of Pathology and Immunology, Washington University School of Medicine in St. Louis, St. Louis, MO, United States; Department of Pathology and Immunology, Washington University School of Medicine in St. Louis, St. Louis, MO, United States; Department of Microbiology, Perelman School of Medicine, University of Pennsylvania, Philadelphia, PA, United States; Department of Pathology and Immunology, Washington University School of Medicine in St. Louis, St. Louis, MO, United States; Department of Pathology and Immunology, Washington University School of Medicine in St. Louis, St. Louis, MO, United States; Department of Pathology and Immunology, Washington University School of Medicine in St. Louis, St. Louis, MO, United States; Department of Microbiology and Immunology, University of Maryland School of Medicine, Baltimore, MD, United States; Center for Systems Immunology, Departments of Immunology and Computational and Systems Biology, University of Pittsburgh, Pittsburgh, PA, United States; Department of Pathology and Immunology, Washington University School of Medicine in St. Louis, St. Louis, MO, United States; Department of Microbiology, Perelman School of Medicine, University of Pennsylvania, Philadelphia, PA, United States; Department of Pathology and Immunology, Washington University School of Medicine in St. Louis, St. Louis, MO, United States; Center for Vaccines and Immunity to Microbial Pathogens, Washington University School of Medicine in St. Louis, St. Louis, MO, United States; Andrew M. and Jane M. Bursky Center for Human Immunology and Immunotherapy Programs, Washington University School of Medicine in St. Louis, St. Louis, MO, United States; Department of Molecular Microbiology, Washington University School of Medicine in St. Louis, St. Louis, MO, United States

**Keywords:** B cell responses, BPTF, germinal center B cells, NURF complex

## Abstract

Germinal centers (GCs) are microanatomical structures in which antigen-specific B cells undergo proliferation, somatic hypermutation, and affinity-based competition to select high-affinity clones that differentiate into memory B cells and plasma cells (PCs). B cell progression through the GC is tightly regulated, and the molecular determinants that modulate GC B cell proliferation and survival are still under investigation. Here, we use a conditional deletion mouse model to demonstrate that bromodomain PHD finger transcription factor (BPTF), a subunit of the nucleosome remodeling factor chromatin remodeling complex, is required for robust GC B cell responses following vaccination. In GC B cells, *Bptf* loss induces a stress-like transcriptional profile and a shift toward PC identity-defining transcriptional programing, although this does not lead to accumulation of functional PCs. Rather, we show that BPTF-deficient GC B cells are prone to cell death. Cumulatively, our data demonstrate that BPTF is necessary for GC B cell maintenance and robust antigen-specific B cell responses.

## Introduction

The humoral immune response is a critical aspect of the host defense against pathogens. Long-lived protective humoral responses are mediated by high-affinity memory B cells (MBCs) and plasma cells (PCs), primarily generated in germinal centers (GCs). The GC is a tightly regulated microanatomical structure within secondary lymphoid tissues where antigen-specific B cells undergo iterative rounds of somatic hypermutation (SHM) and competitive selection to generate pools of high-affinity MBCs and PCs that can protect the host upon subsequent antigen exposure.^1^ Thus, GCs are essential for the development of optimal humoral immune responses to both infection and vaccination.

Upon activation, antigen-specific B cells have multiple potential fates, including differentiation into short-lived plasmablasts (PBs) or entry into a GC.^2^ This decision is modulated by an interconnected network of transcription factors, canonically centered around the mutually antagonistic transcription factors *Bcl6*, which promotes GC B cell transcriptional programming, and *Prdm1* (BLIMP1), which promotes PC fate.^2^

Within the GC, antigen-specific B cells cycle between the dark zone (DZ), where they proliferate and undergo SHM of their B cell receptor (BCR), and the light zone (LZ), where B cell clones compete for survival signals from T follicular helper (Tfh) cells.^3^^,^^4^ SHM within DZ GC B cells can lead to deleterious mutations and subsequent apoptosis, but it can also generate cells with productively mutated BCRs with a range of affinities.^5^ Such cells can cycle to the LZ, where those with high affinity have a competitive advantage in escaping programmed apoptosis by receiving survival signals from Tfh cells.^6^ Upon selection, some LZ GC B cells will differentiate into PCs, concurrent with upregulation of BLIMP1, or MBCs and exit the GC.^1^ Other LZ GC B cells will re-enter the cell cycle and migrate back to the DZ for additional rounds of SHM and proliferation, the magnitude of which is dictated by activity of the transcriptional regulators MYC and mTORC1 induced in the LZ.^1^^,^^7–11^

Given the highly dynamic nature of the GC and the stress imposed by proliferation and SHM on individual cells, numerous factors are required to regulate the maintenance and function of GC B cells. The transcriptional repressor BCL6 is one such master regulator that, among its many roles in GC B cells, represses both DNA damage sensing pathways and the anti-apoptotic gene *Bcl2*, thereby permitting the process of affinity maturation.^1^ BCL6 also represses BLIMP1 expression, thus preventing a switch to PC transcriptional programming within the GC.^2^ In addition to the several key transcriptional regulators identified in GC B cells, there has been a growing appreciation of the drastic changes in the chromatin landscape of B cells as they differentiate into GC B cells.^12^^,^^13^ Indeed, studies that have investigated chromatin remodelers within GC B cells have identified essential roles for members of the SWI/SNF (switch/sucrose nonfermentable) and ISWI (imitation switch) families of chromatin remodelers in the formation and function of GC B cells.^14–18^ However, the founding member of the ISWI family, the nucleosome remodeling factor (NURF), has not yet been independently investigated in GC B cells.

Previous work has demonstrated that the core NURF subunit, bromodomain PHD finger transcription factor (BPTF), is required for stem cell function, the development of embryonic germ layers and thymocytes, and T cell homeostasis.^19–25^ Many studies have also identified a link between elevated BPTF expression and the development or progression of numerous cancers.^26–36^ In conjunction, experimental knockdown of *BPTF* leads to decreased proliferation and/or increased apoptosis in cancer cell lines.^27^^,^^29–35^^,^^37^^,^^38^ Additionally, multiple studies have identified a link between BPTF and transcriptional activity of the oncogene *Myc*, as BPTF is required for optimal recruitment and promoter binding of MYC.^27^^,^^30^^,^^38–41^

Through analysis of transcriptional profiles of vaccine-induced B cell populations in humans, we observed that *BPTF* is highly expressed in GC B cells relative to other B cell populations. This suggests that, among ISWI chromatin remodelers, the NURF complex may play a specific role in GC B cells.^42–44^ To investigate this hypothesis, we generated a conditional deletion *Bptf^flox/^flox* × *Ighg1crecre^/wt^* mouse and utilized a nucleoside-modified messenger RNA (mRNA)–lipid nanoparticle (LNP) immunization model. We show that BPTF is necessary for maintaining a robust GC B cell response, and that loss of *Bptf* results in fewer antigen-specific IgG1^+^ MBCs and PCs at a memory time point. Transcriptional analysis revealed that the *Bptf*-deficient GC B cell pool upregulated *Prdm1* and *Irf4* and downregulated *Bcl6* and *Bach2*, although this did not generate an expanded PC population. Additionally, BPTF-deficient GC B cells shifted away from pathways related to cell cycle progression and instead upregulated pathways and genes associated with cellular stress. Further investigation revealed that the BPTF-deficient GC B cell compartment had elevated levels of mitochondria-derived reactive oxygen species (ROS), a lower frequency of LZ cells in the S phase of the cell cycle, and increased caspase activation. Together, these data suggest that the loss of BPTF alters GC B cell identity and triggers a cascade of stress responses that can ultimately result in cell death.

## Methods

### Human samples for in vitro culture

Blood product was flushed from leukoreduction system chambers collected from healthy platelet donors using phosphate-buffered saline (PBS) containing 2% fetal bovine serum (FBS) (Corning) and 2 mM EDTA (Corning) (P2). Peripheral blood mononuclear cells (PBMCs) were isolated by density gradient centrifugation. B cells were enriched from PBMCs using a pan-B cell enrichment kit (StemCell; Cat#19554) according to the manufacturer’s instructions. Enriched B cells were resuspended at a concentration of 600,000 cells/mL in B cell media (BCM) (RPMI 1640 containing L-glutamine [Corning] with 10% FBS [Corning], 10 mM HEPES [Corning], 100 units/mL penicillin + 100 µg/mL streptomycin [Gibco], and 5.5 × 10^−5^ M 2-mercaptoethanol [Sigma-Aldrich]) and 300,000 cells were added to each well in a 24-well plate. A stimulant cocktail containing 1 µg/mL R848 (Sigma-Aldrich; Cat#SML0196), 10 µg/mL anti-IgA + IgG + IgM (H + L) (Jackson ImmunoResearch; Cat#109-005-064), and 0.1 µg/mL CD40L (Cell Sciences; Cat#CRM030) was added immediately upon plating. Additionally, each well was treated with either 5 µM of AU1 (Aobious; Cat#AOB0117) or an equivalent volume of dimethyl sulfoxide (DMSO) (Sigma-Aldrich). Cells were incubated at 37 °C in 5% CO_2_ and harvested 14 to 62 h later by collecting the BCM and rinsing the well once with P2.

### Single-cell RNA-sequencing

Newly sequenced BCR and transcriptomics data from IgD^lo^ B cells from blood collected at week 16 from 4 participants and at week 29 from 1 participant postimmunization were combined with previously reported BCR and transcriptomics data and reprocessed together as previously described.^43^^,^^44^ Heavy chain–based clonal inference was performed using a CDR3 nucleotide dissimilarity cutoff of 0.15 normalized Hamming distance. A B cell clone was designated SARS-CoV-2 spike (S) specific if the clone contained any BCR sequence corresponding to a recombinant monoclonal antibody that tested positive for S-binding. With respect to the transcriptomics data, after quality control, the feature matrix contained measurements for 15,884 genes across 562,296 cells from 72 single-cell samples. Single-cell gene expression analysis was performed as previously described, with adaptations where applicable for the current data.^44^ Overall clusters were identified using Leiden graph-clustering with resolution 0.16. A total of 98 cells of blood origin that colocalized with the follicular dendritic cell cluster were removed. Cells from the overall B cell cluster were further clustered to identify B cell subsets using Leiden graph clustering with a resolution of 0.55. One cluster containing 2,025 cells showed no discernible expression pattern of the marker genes used and was thus labeled unassigned and subsequently excluded from the final B cell clustering. Following gene expression–based B cell subset annotation, cells were subset to those present in S+ B cell clones. Expression level of *BPTF* was then visualized, faceting by B cell subset.

### Mice

All experiments were approved by the Washington University in St. Louis Institutional Animal Care and Use Committee. The following mouse strain was purchased from the Jackson Laboratory: B6.129P2(Cg)-*Ighg1^tm1(cre)Cgn^*/J (JAX Strain #010611). Floxed *Bptf* mice were designed and generated by the Genome Engineering and Stem Cell Center at Washington University in St. Louis. Briefly, guide RNAs (gRNAs) were designed in introns 1 and 2, floxing about a 1.15 kb region. Synthetic gRNAs were ordered from Integrated DNA Technologies, the one in intron 1 with spacer sequence (protospacer adjacent motif) 5′- tttgctatgtcacaattctg(ggg), and in intron 2, 5′-gacttgagatggaaggaatg(cgg). A single stranded oligo DNA donor (ssODN) carrying loxP in the CRISPR cleavage site flanked by short homology arms was used as a donor template with each gRNA. The ssODN for intron 1 has the sequences of 5′- g*a*tcagaggttagagtatcgctttgtggtagggcacttacctagtggaatcagccccagGGATCCATAACTTCGTATAGCATACATTATACGAAGTTATaattgtgacatagcaaaagtaacatcagcagggtgtgagggcacagcctttaatccca*g*c and for intron 2 has the sequences of 5′- c*t*aaggcacatgaactcaccttacatcacaatttctaaaatgccgtattaatgcccgcatGGATCCATAACTTCGTATAGCATACATTATACGAAGTTATtccttccatctcaagtctttaactacaattcattattaaaccacacgtttaataatt*g*c (*stands for a phosphorothioate bond). Both ssODNs were ordered as Ultramers from IDT. The gRNA/Cas9 protein complexes were transfected along with the ssODNs into N2A cells and validated by next-generation sequencing for efficient incorporation of cleavage and insertion of loxP before the reagents were used in for generation of transgenic mice. Gene editing was done at the Transgenic, Knockout and Microinjection Core at Washington University in St. Louis. gRNA/Cas9 complex along with both ssODNs were electroporated into single-cell embryos, which were then transferred into pseudopregnant recipients for creation of the founder animals.^45^ Live births were genotyped as during validation using next-generation sequencing. Wild-type (WT) × *Ighg1*cre^cre/wt^ control mice were crossed into the *Bptf* strain and bred in-house. Both male and female mice (6–12 wk old) were used for experiments.

### mRNA-LNP formulation

SARS-CoV2 S mRNA-LNP vaccines were manufactured as described.^46^ mRNA encodes the full-length SARS-CoV-2 WA1 S with ablated furin cleavage site.^47^^,^^48^ Vaccines were stored at −80 °C.

### Immunizations

SARS-CoV-2 S mRNA-LNP aliquots were thawed on ice and diluted in cold PBS (Leinco Technologies) before being administered intramuscularly in a 50 µL volume. Mice were immunized with 5 to 10 µg of vaccine, either unilaterally or bilaterally.

### Tissue processing

Spleens and draining lymph nodes (dLNs) (inguinal and iliac) were harvested and forced through a 70 µm cell strainer into P2 or RPMI 1640 containing L-glutamine (Corning) with 2% FBS and 1,000 units/mL penicillin/1,000 µg/mL streptomycin (Gibco) (R2) to make single-cell suspensions. LNs were washed once then resuspended in P2 or R2. Spleens were treated with 1× RBC Lysis Buffer (BioLegend; Cat#420302) and washed before resuspension.

### Mouse in vitro culture

A spleen from a naïve WT mouse was processed in P2 as described previously in sterile conditions. B cells were negatively enriched from the splenocyte pool using the EasySep Mouse B cell Isolation Kit (StemCell; Cat#19854) according to kit instructions. B cells were resuspended in cold BCM at 600,000 cells per mL. A PC-skewing stimulant cocktail containing 15 ng/mL IL-4 (R&D Systems; Cat#404-ML-010), 1 µg/mL R848 (Sigma-Aldrich; Cat#SML0196), and 0.1 µg/mL CD40L (Cell Sciences; Cat#CRM030) was added to the B cells and 300,000 cells were plated per well of a 24-well plate. Additionally, each well was treated with either 5 µM of AU1 (Aobious; Cat#AOB0117) or an equivalent volume of DMSO (Sigma-Aldrich). Cells were incubated at 37 °C in 5% CO_2_ and harvested 24 or 48 h later by collecting the BCM and rinsing the well once with P2.

### Antigens

Recombinant soluble SARS-CoV-2 S protein was produced as previously described.^49^ In brief, mammalian cell codon-optimized nucleotide sequences coding for the soluble form of S (GenBank: MN908947.3, amino acids 1–1,213) was modified to remove the polybasic cleavage site (RRAR to A) and add two stabilizing proline mutations (K986P and V987P, WT numbering), then cloned into the mammalian expression vector pCAGGS.^50^ For expression of Avi-tagged S, the CDS of pCAGGS vector containing the S sequence was modified to encode a 3′ Avitag insert after the 6×His tag (5′-His tag-GGCTCCGGGCTGAACGACATCTTCGAAGCCCAGAAGATTGAGTGGCATGAG-Stop-3′; HHHHHHGSGLNDIFEAQKIEWHE-). Expi293F cells (Thermo Fisher Scientific) were transfected with purified plasmid using the ExpiFectamine 293 Transfection Kit (Thermo Fisher Scientific) and incubated at 37 °C (5% CO_2_) for 4 to 6 d. Supernatants from transfected cells were collected and recombinant proteins were purified using Ni-NTA agarose (Thermo Fisher Scientific), buffer exchanged into PBS, and concentrated using Amicon Ultracel centrifugal filters (EMD Millipore). After purification, S-Avitag substrates were diluted to 40 µM and incubated for 1 h at 30 °C with 15 µg ml^−1^ BirA enzyme (Avidity) in 0.05 M bicine buffer at pH 8.3 supplemented with 10 mM ATP, 10 mM magnesium acetate, and 50 µM biotin. The protein was then concentrated and buffer-exchanged into PBS using a 100 kDa Amicon Ultra centrifugal filter (EMD Millipore). S protein was stored at −80 °C. To make fluorescent flow cytometry probes, avi-tagged S was incubated with a 1.04-fold molar excess of APC-Fire 750-Streptavidin (BioLegend; Cat#405250) or BV650-Streptavidin (BioLegend; Cat#405232). Specifically, one-third of the avi-tagged S protein was added to the total streptavidin volume at 20-minute intervals. Immediately after the final S addition, a 6-fold molar excess of d-biotin was added. A 100% volume of glycerol was added and mixed thoroughly by pipetting. Probes were stored at −20 °C.

### Flow cytometry

All blocking, staining, and fixation steps were performed in a volume of 25 µL per 1 × 10^6^ cells. Freshly harvested cells were incubated for 20 min on ice with CD16/32 (1:100, 93; BioLegend; Cat#101302) in P2, then washed once. A master mix of S probes, antibodies targeting surface markers, and a live/dead stain was made in Brilliant Stain Buffer (BD Horizon; Cat #563794) or 1:10 True-Stain Multi-Fluor Buffer (BioLegend; Cat#426106) in P2. Anti-human antibodies used include CD19-PE (1:200, HIB19; BioLegend; Cat#302254), CD3-SparkUV387 (1:200, HIB19; BioLegend; Cat#300338), and CD14-SparkUV387 (1:200, S18004B; BioLegend; Cat#399216). Anti-mouse antibodies used include CD138-BV421 (1:200, 281-2; BioLegend; Cat#142508), CD71-SB436 (1:50, R17217; Invitrogen; Cat#62-0711-82), IgG1-BV510 (1:100, RMG1-1; BioLegend; Cat#406621), CD4-BV570 (1:200, RM4-5; BioLegend; Cat#100542), CXCR4-BV605 (1:50, L276F12; BioLegend; Cat#146519), IgD-BV711 (1:200, 11-26c.2a; BioLegend; Cat#405731), CD19-BV750 (1:200, 6D5; BioLegend; Cat #115561), GL7-PerCP-Cy5.5 (1:50, GL7; BioLegend; Cat#144610), CD95-PE (1:200, Jo2; BD Biosciences; Cat#554258), CD38-PE Dazzle 594 (1:400, 90; BioLegend; Cat#102730), CD86-PE-Cy5 (1:400, GL-1; BioLegend; Cat#105016), IgM-PE-Cy7 (1:400, RMM-1; BioLegend; Cat#406514), PD-1-BUV737 (1:100, J43; Invitrogen; Cat#367-9985-82), CXCR5-BV421 (1:50, L138D7; BioLegend; Cat#145512), CD138-PE-Fire 810 (1:400, 281-2; BioLegend; Cat#142545), CXCR4-PE-Vio615 (1:50, REA107; Milltenyi Biotec; Cat#130-107-610), and CD38-FITC (1:400, 90; BioLegend; Cat#102705). Viability stains used include Zombie NIR Fixable Viability Kit (BioLegend; Cat#423106) and Zombie Aqua Fixable Viability Kit (BioLegend; Cat#423102). Cells were suspended in the master mix and incubated on ice for 30 min. After two P2 washes, cells were sorted (see the following); further stained using MitoSOX, caspase-3/7, or EdU kits (see the following); permeabilized and fixed for intranuclear staining; or fixed in 2% paraformaldehyde (PFA) (diluted in P2 from 4% PFA in PBS) for 20 min on ice. Cell fixed with 2% PFA were washed with P2 and acquired the next day. For intranuclear staining, cells were resuspended in 1× True-Nuclear Transcription Factor Buffer Set (BioLegend; Cat#424401) and incubated for 50 to 60 min at room temperature. Cells were washed at least twice with the 1× Perm Buffer provided in the kit then resuspended in a master mix including BCL6-RB780 (1:400, K112-91; BD Biosciences; Cat#569142) and BLIMP1-R718 (1:100, 6D3; BD Biosciences; Cat#567764). Cells were stained overnight at 4 °C. All samples were acquired on a Cytek Aurora using SpectroFlo v3.3 and analyzed using FlowJo v10 (TreeStar).

### Cell sorting

For cells to be used for bulk RNA sequencing or quantitative polymerase chain reaction (PCR), *Bptf*^wt/wt or fl/fl^ × *Ighg1*cre^cre/wt^ mice were immunized with 10 µg of SARS-CoV-2 S mRNA-LNP vaccine. Spleens and dLNs were collected and prepared as described previously. Cells from *Bptf*^wt/wt or fl/fl^ × *Ighg1*cre^cre/wt^ mice were pooled where indicated in the figure legends. B cells were enriched using the StemCell negative B cell enrichment kit (#19854) according to manufacturer instructions. Cells were stained as described above with surface markers. Cells were washed with P2 and sorted into RLT Plus buffer (Qiagen; Cat#80204) supplemented with 143 mM 2-mercaptoethanol (Sigma-Aldrich) using a Bigfoot (Invitrogen).

RNA and DNA were isolated with the AllPrep DNA/RNA kit (Qiagen; Cat#80204) according to the manufacturer’s instructions and stored at −80 °C.

### EdU detection

EdU (MilliporeSigma; Cat#900584) was reconstituted in DMSO (Sigma-Aldrich) at 200 mg/mL and stored at −20 °C. Mice were intravenously injected with 2 mg EdU (diluted in 200 µl of PBS) 1 h before harvest. Cells were harvested and stained for surface antigens as described above. EdU was detected using the Click-iT Plus EdU AF647 Flow Cytometry kit (Invitrogen; Cat#C10634) following manufacturer instructions except all steps were performed at a concentration of 4 × 10^6^ cells/mL and the protocol was paused overnight after permeabilization. After washing off the reaction cocktail, cells were placed on ice and immediately acquired by flow cytometry.

### Caspase-3/7 staining

Cells were harvested and stained for surface antigens as described previously. After washing, cells were resuspended at a concentration of 1.6 × 10^7^ cells/mL in P2 and stained with 500 nM CellEvent Caspase 3/7 (Thermo Fisher Scientific; Cat#C10427) for 45 min at room temperature. Samples were put on ice and immediately analyzed by flow cytometry.

### MitoSOX staining

A 1 mM stock solution of MitoSOX Green reagent (Thermo Fisher Scientific; Cat#M36006) was prepared according to the manufacturer’s instructions. Cells were surface stained as described previously. After washing, cells were resuspended at a concentration of 5 × 10^6^/mL in P2 and stained with 1 µM of the MitoSOX Green reagent for 1 h at room temperature. Cells were washed only once before resuspension then put on ice and immediately analyzed by flow cytometry.

### Quantitative real-time PCR

Isolated RNA was reverse transcribed into complementary DNA (cDNA) using the High-Capacity cDNA Reverse Transcription kit (Applied Biosystems; Cat#4368814) according to the manufacturer’s protocol. Quantitative PCR was performed using Power SYBR Green PCR Master Mix (Applied Biosystems; Cat#4367659) and the following primers: BPTF sense 5′-CCAGCACAGAGAAGACCATGATAAG-3′; BPTF antisense 5′-CGTGAACAGACCCGTTCCATT-3′; β-actin F 5′-TGAGCTGCGTTTTACACCCT-3′; β-actin R 5′-GCCTTCACCGTTCCAGTTTT-3′ *Bptf* expression levels were calculated using Cq values normalized to β-actin.

### Bulk RNA sequencing (library preparation and analysis)

Total RNA integrity was determined using Agilent Bioanalyzer or 4200 TapeStation. Library preparation was performed with 3 to 10 ng of total RNA with a Bioanalyzer RIN score >8.0. ds-cDNA was prepared using the SMARTer Ultra Low RNA kit for Illumina Sequencing (Takara-Clontech) per the manufacturer’s protocol. cDNA was fragmented using a Covaris E220 sonicator using peak incident power of 18, 20% duty factor, and 50 cycles per burst for 120 s. cDNA was blunt-ended, had an A base added to the 3′ ends, and then had Illumina sequencing adapters ligated to the ends. Ligated fragments were then amplified for 12–15 cycles using primers incorporating unique dual index tags. Fragments were sequenced on an Illumina NovaSeq X Plus using paired-end reads extending 150 bases. Basecalls and demultiplexing were performed with Illumina’s bcl2fastq software with a maximum of 1 mismatch in the indexing read. RNA sequencing reads were then aligned to the Ensembl GRCm39.113 primary assembly with STAR version 2.7.11b. Gene counts were derived from the number of uniquely aligned unambiguous fragments by Subread: featureCount version 2.0.8. Isoform expression of known Ensembl transcripts were quantified with Salmon version 1.10.0. Sequencing performance was assessed for the total number of aligned reads, total number of uniquely aligned reads, and features detected. The ribosomal fraction, known junction saturation, and read distribution over known gene models were quantified with RSeQC version 5.04.

### Differential expression analysis

Bulk RNA sequencing count data were analyzed in RStudio (version 4.5.0) using the DESeq2 package (version 1.48.1). After importing the count matrix, genes were retained if they showed at least 5 read counts in a minimum of 3 samples. Sample metadata were parsed to construct the experimental design matrix, with genotype conditional knockout (cKO) as the main variable of interest and WT set as the reference condition. A DESeq2 dataset was created using rounded raw counts and processed using the standard workflow by fitting of negative binomial generalized linear models (DESeq). Differential expression testing was performed for the contrast cKO vs WT samples. Log_2_ fold-changes were shrunk using the ashr method to improve effect size estimation for low-abundance genes. *P* values were adjusted using the Benjamini–Hochberg procedure to control the false discovery rate (FDR). Genes were considered significantly differentially expressed if they had an adjusted *P* value <0.05 and |log_2_(fold change)| >0.6. For the genes highlighted in the volcano plots, a bespoke unfolded protein response (UPR)/endoplasmic reticulum (ER) stress list was curated by combining the gene lists (mouse) of UPR from Qiagen GeneGlobe, Reactome UPR from gene set enrichment analysis (GSEA), ER UPR from MGI Gene Ontology (GO) annotations (GO: 0030968), Hallmark UPR from GSEA, and the following genes from the GO biological process response to ER stress (GSEA): *Jagn1*, *Ufc1*, *Sesn2*, *Man1c1*, *Rcn3*, *Nod1*, *Pml*, *Niban1*, *Trib3*, *Nupr1*, and *Pdia4*.

### Principal component analysis

Normalized expression values were obtained by applying the variance-stabilizing transformation (VST) to the DESeq2 object created for the cKO vs WT contrast in RStudio (version 4.5.0). For principal component analysis (PCA), we restricted the matrix to genes meeting the differential-expression criteria. The resulting VST matrix was subset to these significant genes, transposed to samples by genes, and subjected to PCA using the prcomp function. The percentage of variance explained by each principal component (PC) was calculated from the PCA object. Sample coordinates on PC1 and PC2 were visualized, displaying genotype groups cKO vs WT and sample labels.

### Gene set enrichment analysis

GSEA was performed in RStudio (version 4.5.0) using the fgsea package together with msigdbr to access the Hallmark gene sets from MSigDB (Molecular Signatures Database) (“H” collection; *Mus musculus*). Genes were first ranked by their log_2_ fold change values from the DESeq2 differential expression results, forming a continuous ranking metric. Duplicate gene entries were collapsed by retaining the one with the largest absolute fold change. The Hallmark mouse gene sets were obtained using msigdbr, and pathways were defined as sets containing between 15 and 500 genes. Enrichment analysis was then performed using the fgsea() function, with 10,000 permutations to compute normalized enrichment scores, nominal *P* values, and adjusted *P* values (FDR). Significantly enriched pathways were defined as those with FDR <0.05. Leading-edge genes (i.e. the subset contributing most to the enrichment score) were extracted for visualization.

### Immunofluorescent microscopy

After harvest, half of the spleen was taken and submerged in 1% PFA (in PBS) for 1 h at room temperature. After a PBS rinse, tissues were transferred to 30% sucrose (in water) and placed at 4 °C overnight. Tissues were embedded in Optimal Cutting Temperature (O.C.T) embedding medium and frozen using a dry ice–ethanol bath. The 10-µm-thick sections were cut onto Superfrost Plus slides and stored at −80 °C. A total of 1 to 5 sections were taken per mouse, each separated by at least 100 µm. Slides were dried overnight at room temperature before staining. Slides were fixed for 15 min at −20 °C in prechilled 10% neutral buffered formalin fixative (Thermo Fisher Scientific) then washed with PBS and blocked with 1% bovine serum albumin (BSA) (in PBS) for 2 h at room temperature. After removing the BSA, sections were covered with a droplet of staining mix (in 1% BSA): IgD BV421 (1:100, 11-26c.2a; BioLegend; Cat#405725), CD4 AF594 (1:50, GK1.5; BioLegend; Cat#100446), and GL7 AF647 (1:100, GL7; BioLegend; Cat#144606). Slides were stained in humidity for 16 to 24 h at 4 °C. After PBS washes, slides were manually dried and immediately mounted with Vectashield Vibrance Antifade Mounting Medium (Vector Laboratories; Cat#H-1700-2) and #1 thickness coverslips and sealed with nail polish. Slides were stored at 4 °C before imaging with a Zeiss AxioScan 7 Slide Scanner at 20×/0.8. All samples from each individual experiment were stained and imaged together. Images were analyzed using Fiji v2.9.0 (ImageJ; National Institutes of Health); the GL7 brightness threshold was adjusted and kept consistent between all sections from the same experiment. Germinal center areas were measured using the “Analyze Particles” function with a minimum gate size set to 800 µm^2^.

### ELISpot

After ethanol activation, plates (Sigma-Aldrich; Cat#MSIPN4W50) were coated with 100 µL of S protein at 1 µg/mL at 4 °C overnight. Plates were washed with PBS + 0.05% Tween 20 (PBST) (Sigma-Aldrich), then blocked with 200 µL RPMI 1640 containing L-glutamine (Corning) with 10% FBS (Corning) and 1,000 units/mL penicillin/1,000 µg/mL streptomycin (Gibco) (R10) for at least 2 h at 37 °C (9% CO_2_). After removing the blocking buffer, 1 × 10^6^ cells were added to the first well in a total of 200 µL of R10 and a 3-fold dilution was performed. Cells were incubated for 4 h at 37 °C (9% CO_2_) then washed with PBS followed by PBST. A total of 100 µL of anti-mouse IgG1-biotin (Southern Biotech; Cat#1070-08) was added to each well at a concentration of 0.5 µg/mL in P2 and incubated overnight at 4 °C. Plates were washed with PBST then incubated for 1.5 h at room temperature with 100 µL of horseradish peroxidase conjugated streptavidin (Jackson ImmunoResearch; Cat#016-030-084) at a concentration of 0.2 µg/mL in P2. After washing with PBST then PBS, the plate was developed with 100 µL/well of developing buffer (10 mL of 0.1 M Sodium Acetate Buffer + 150 µL of 20 mg/mL AEC, syringe filtered with a 0.45 µm filter, then added 100 µL H_2_O_2_). After spots developed, the buffer was discarded and the plate was rinsed with DI water. Plates were imaged using an ELISpot plate reader (Cellular Technology) and spots were manually counted.

### ELISA

Plates (Greiner Cat#655085) were coated overnight at 4 °C with 100 µL of 1 µg/mL S protein in PBS. Plates were washed with PBST then blocked with 200 µL blocking buffer (PBST + 10% FBS) for 1.5 h at room temperature. After removing the blocking buffer, serum samples diluted in blocking buffer were added to wells. A 3-fold dilution was performed, for a total volume of 50 µL/well. Plates were incubated for 1.5 h at room temperature then washed with PBST. A total of 100 µL of IgG1-horseradish peroxidase (1:1,000; Southern Biotech; Cat#1070-05) in blocking buffer was added for 1 h before washing with PBST then PBS. A total of 100 µL of substrate solution (1 tablet of OPD (4 mg) [Sigma-Aldrich; Cat#P8787] + 10 mL phosphate citrate buffer [Sigma-Aldrich; Cat#P4809] + 0.01% H_2_O_2_) was added to each well and allowed to develop for 5 min before the reaction was stopped with 100 µL 1 M HCl. Absorbance was measured at 490 nm.

## Results

### BPTF is upregulated in human GC B cells

Using previously generated human datasets,^43^^,^^44^ we identified *BPTF* as a gene of interest in GC B cells. *BPTF* is highly expressed in antigen-specific GC B cells following vaccination relative to PBs, MBCs, and naïve B cells (Fig. S1A). Numerous studies have linked *BPTF* to cellular proliferation and survival, primarily in the setting of cancer, but its role in noncancerous mature B cells is less understood.^27^^,^^29–35^^,^^37^^,^^38^ To investigate this, we enriched primary human B cells from blood and cultured them for 2.5 d with a small molecule inhibitor of BPTF (AU1^51^) CD40L, R848, and anti-IgM + IgG + IgA. Between the 0.5- and 2.5-d time points after plating, cells treated with the DMSO vehicle control on average expanded 1.6-fold *in vitro*, whereas we observed no expansion of cells treated with AU1 (Fig. S1B). These data suggest that BPTF is necessary for the proliferation and/or survival of primary B cells *in vitro*.

### BPTF is necessary for a robust vaccine-induced GC B cell response

**Figure 1:**
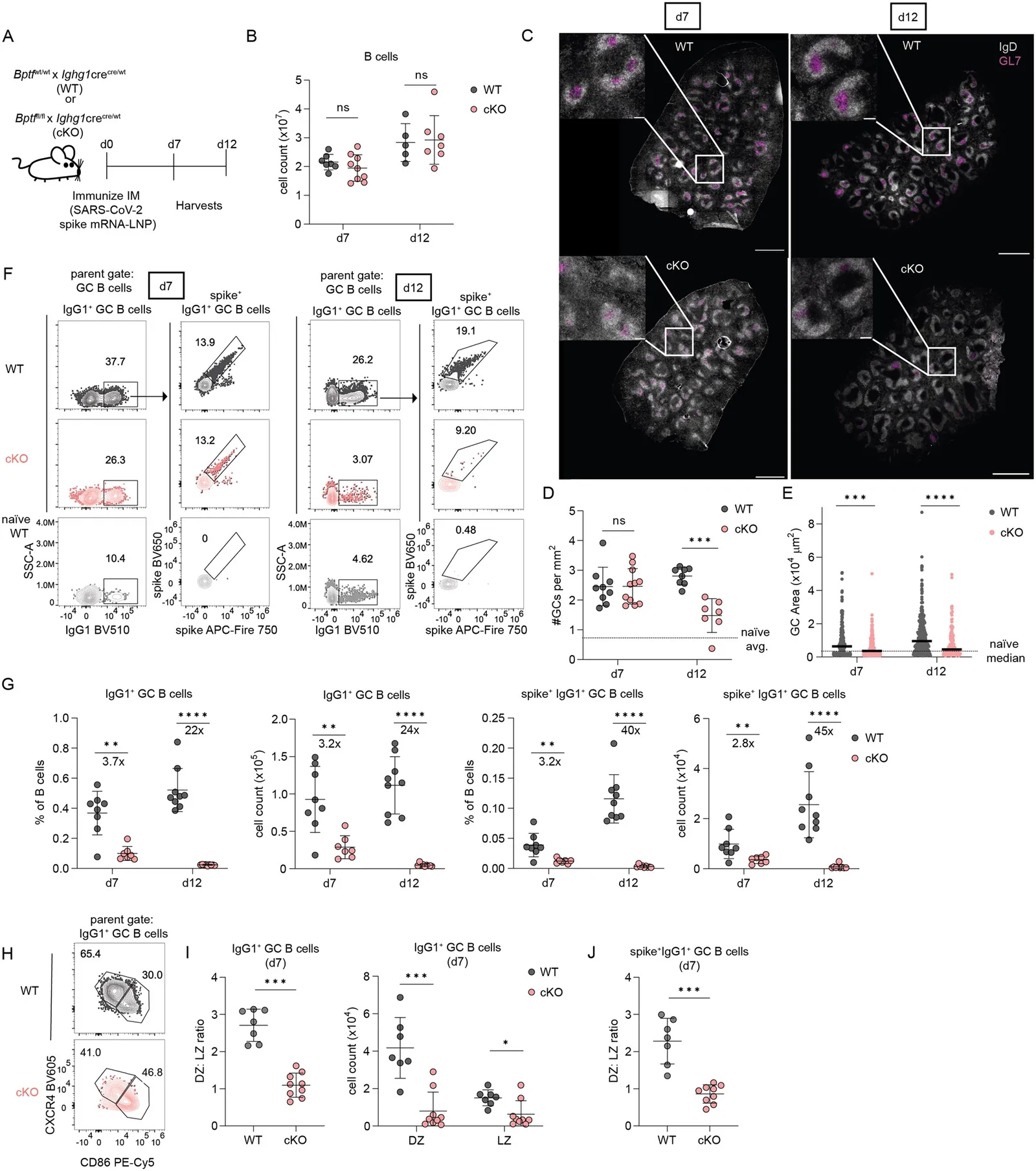
BPTF is necessary for robust GC B cell responses following vaccination. (A) Experimental design. *Bptf*^wt/wt or fl/fl^ × *Ighg1*cre^cre/wt^ mice (WT or cKO) were immunized intramuscularly with a single dose of 5 to 10 µg of SARS-CoV-2 S mRNA-LNP vaccine. Spleens, dLNs, and serum were harvested either 7 or 12 d postimmunization. (B) Number of splenic B cells (CD19^+^CD4^−^) 7 and 12 d postimmunization as measured by flow cytometry. n = 5–9 mice per group. Day 7 (d7) representative of 5 and d12 representative of 3 independent experiments. (C) Representative immunofluorescence microscopy images of d7 and d12 spleen slices. IgD is gray, GL7 is magenta. Scale bars in full images are 1 mm and scale bars in inlay images are 100µm. (D) Quantification of GC structures normalized as GCs per mm^2^. A GCs/mm^2^ number was calculated for each spleen slice, and 1 individual data point represents the average of all slices analyzed for 1 mouse (average of 2.7 slices). Dotted line represents the average number of GCs/mm^2^ in 3 naïve mice. GC structures were defined as a GL7 cluster >800µm^2^ within an IgD^+^ follicle. Pooled from 4 independent experiments (2 per time point). (E) Areas of GC structures pooled from 4 or 5 mice per group. If multiple spleen slices were analyzed for 1 mouse, only GC areas from the slice with the most GCs were used. The dotted line represents the median area of GCs in 3 naïve mice. Number of GC structures analyzed: d7 WT n = 276, d7 cKO n = 228, d12 WT n = 304, and d12 cKO n = 134. Each time point is representative of 2 independent experiments. (F) Representative flow plots identifying S^+^IgG1^+^ GC B cells (CD19^+^CD4^−^CD138^−^GL7^+^Fas^+^CD38^lo^IgD^−^IgG1^+^S^+^) in the spleen at d7 and d12. (G) Quantification of IgG1^+^ GC B cells and S^+^IgG1^+^ GC B cells in the spleen as gated in panel F. n = 7–9 mice per group. d7 representative of 5 and day 12 representative of 3 independent experiments. (H) Representative flow plots showing DZ (CXCR4^+^CD86^−^) and LZ (CXCR4^−^CD86^+^) staining within the IgG1^+^ GC B cell compartment in a d7 spleen. (I) Ratio of DZ to LZ IgG1^+^ GC B cells and number in the spleen at d7 as gated in panel H. n = 7–9 mice per group. Representative of 5 independent experiments. (J) Ratio of DZ to LZ cells within the S^+^IgG1^+^ GC B cell compartment in the spleen at d7. n = 7–9 mice per group. Representative of 5 independent experiments. *P < 0.05, **P < 0.01, ***P < 0.001, ****P < 0.0001; Mann-Whitney test. WT mice are represented by gray circles and cKO mice are represented by pink circles. Each symbol represents 1 animal. ns, not significant; SSC-A, side scatter area.

To determine whether BPTF plays a functional role in GC B cells, we designed a transgenic mouse with loxP sites flanking *Bptf* exon 2 (Fig. S2A). Exon 2 is present in the majority of BPTF isoforms, and previous characterization of an independently designed *Bptf*-deletion mouse confirmed that *Bptf* mRNA from *Bptf*^ΔExon2^ mice was spliced out of frame.^22^ This suggested that exon 2 is a viable target for knockout of the full-length BPTF protein. Subsequent cross of a *Bptf*^floxed^ mouse designed alongside the *Bptf^Δ^*^Exon2^ mouse to an *Mb1-*Cre resulted in significantly impaired B cell development, suggesting that B cells require BPTF before GC B cell differentiation.^41^ Thus, to study the role of BPTF in mature B cells only after activation, we crossed our *Bptf*^floxed^ mice to *Ighg1-*Cre mice, in which IgG1^+^ B cells delete *Bptf* upon Cre-mediated recombination.^52^ Although the *Ighg1*-Cre may also impact IgG1^−^ GC B cells due to a combination of IgG1^+^ cells switching isotypes after Cre activity and germline transcription of Cγ1 in non-IgG1 cells, we focused our analysis specifically on IgG1^+^ GC B cells.^52^ To induce a robust primary GC, we immunized these mice (*Bptf*^wt/wt or flox/flox^ × *Ighg1*cre^cre/wt^; “WT” or “cKO”) with a single dose of a SARS-CoV-2 S mRNA-LNP vaccine. In this model, the GC compartment is approximately 30% to 40% IgG1^+^ in the spleen and 20% to 30% IgG1^+^ in the dLNs. We harvested tissues on day 7 postimmunization, a relatively early GC time point, or day 12, which is the peak of the GC in the spleen (Fig. 1A). To validate *Bptf* deletion in this system, we sorted IgG1^+^ GC B cells on day 7 or 12 and confirmed decreased *Bptf* expression by quantitative polymerase chain reaction and excision of exon 2 at the DNA level (Fig. S2C, D).

Following vaccination, we observed no difference in the number of total B cells (CD19^+^CD4^−^) in the spleen between WT and cKO mice by flow cytometry (Fig. 1B). However, immunofluorescence microscopy of the spleen revealed a severely impaired GC response at day 12 postimmunization (Fig. 1C). WT and cKO mice had the same number of GC structures at day 7, but those in cKO mice were on average smaller (Fig. 1D, E). At day 12, while the number of GC structures in the spleens of WT mice had increased relative to day 7, the number in cKO mice had decreased, and the structures present were still significantly smaller than those in WT mice (Fig. 1D, E). Next, we used flow cytometry to characterize the antigen-specific IgG1^+^ GC B cell response specifically (Fig. 1F; Fig. S2B). In both the spleen and dLNs of cKO mice, we observed significantly fewer total and S-specific IgG1^+^ GC B cells at both day 7 and day 12 (Fig. 1G; Fig. S2E). Although we observed relatively high frequencies of S-labeled IgG1^+^ GC B cells in the dLNs, we ultimately recovered very few cells (Fig. S2E). Thus, we focused our subsequent analyses on the spleen. Despite the substantial defect in the GC response we observed in cKO mice, it appears that 1 copy of the *Bptf* allele is sufficient for a robust GC B cell response; *Bptf*^fl/wt^ × *Ighg1*cre^cre/wt^ mice had a comparable frequency of S^+^IgG1^+^ GC B cells to WT mice at day 12 (Fig. S2F). We observed no difference in Tfh cell frequency at day 12, perhaps due to sufficient GC B cell interactions at early time points and limited expansion of the Tfh cell compartment relative to naïve mice (Fig. S2G, H).

At day 7, prior to the complete collapse of the GC, we observed a decrease in the frequency of DZ (CXCR4^+^CD86^−^) cells within the total and S-specific IgG1^+^ GC compartment of cKO mice. This corresponded to an increase in the frequency, but not the number, of LZ (CXCR4^−^CD86^+^) cells and cells that fell into neither the DZ compartment nor the LZ compartment (CXCR4^−^CD86^−^) (Fig. 1H–J). Although the GCs we identified through immunofluorescence microscopy were not solely IgG1 expressing, the number of GC structures combined with the quantification of S^+^IgG1^+^ GC B cells at day 7 together suggests that BPTF-deficient IgG1^+^ cells have the capacity to form and/or enter GCs. Although we cannot rule out that these abilities may be impaired, the collapse of the IgG1^+^ GC between days 7 and 12 and the skewed DZ:LZ ratio led us to conclude that BPTF is necessary for the maintenance of GC B cells.

### BPTF cKO mice have fewer MBCs and PCs at a memory time point

To investigate the potential consequences of the impaired GC response we observe in cKO mice, we immunized a cohort of WT and cKO mice and harvested serum, bone marrow, and spleens 3 mo later to characterize memory compartments (Fig. S3A). In accordance with the reduced GC response, cKO mice had significantly fewer S^+^IgG1^+^ bone marrow antibody-secreting cells (ASCs) at 3 mo postimmunization compared with WT mice (Fig. S3B, C) and lower S-specific IgG1 serum antibody titers (Fig. S3D). Next, we quantified S-specific MBCs in the spleen and found that cKO mice had significantly fewer S^+^IgG1^+^ MBCs than WT mice (Fig. S3E, F) but comparable numbers of S^+^IgM^+^ MBCs (Fig. S3G). It is possible that at least some S^+^IgG1^+^ MBCs and bone marrow PCs we observe at the 3-mo time point are cells with incomplete BPTF deletion, as we see some evidence of that in the IgG1^+^ GC B cell compartment at day 12 (Fig. S2D). However, if they are BPTF-deficient, we cannot rule out that BPTF plays an independent role in maintaining MBCs and PCs without an inducible deletion model. However, based on the decreased magnitude of the GC we observed at earlier time points, we hypothesize that the reduced number of these populations at least partially reflects diminished GC responses in cKO mice rather than solely MBC- or bone marrow PC–specific functions of BPTF.

### BPTF alters the transcriptional profile of GC B cells

**Figure 2:**
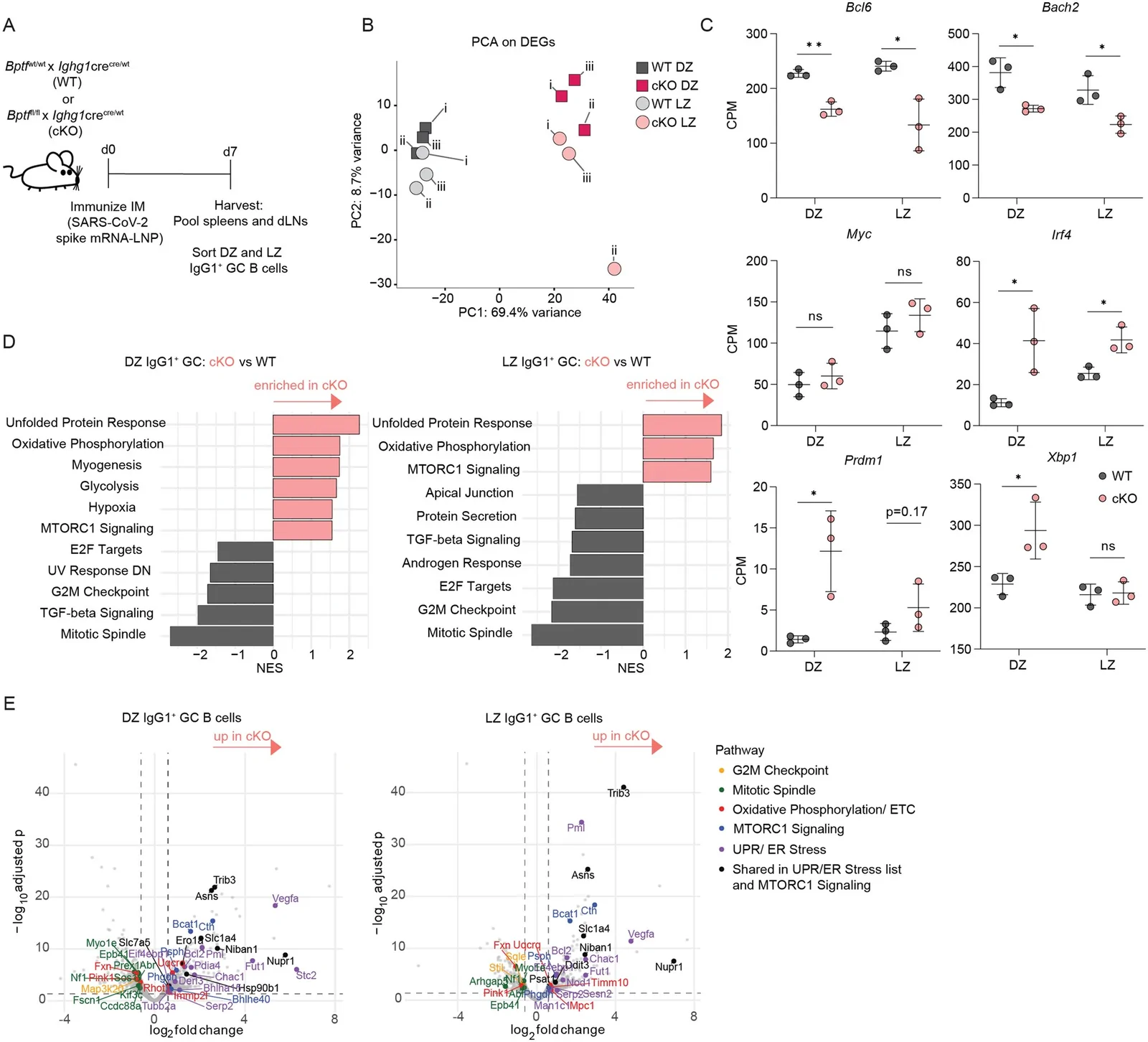
BPTF-deficient GC B cells are enriched for genes associated with PC identity and cellular stress. (A) Experimental design. *Bptf*^wt/wt or fl/fl^ × *Ighg1*cre^cre/wt^ mice (WT or cKO) were immunized intramuscularly with a single dose of 10 µg of SARS-CoV-2 S mRNA-LNP vaccine. Spleens and dLNs harvested 7 d postimmunization. DZ and LZ IgG1^+^ GC B cells were harvested from pools of all WT or cKO tissues. 3 independent experiments with 4 to 7 WT mice and 7 to 15 cKO mice were performed. (B) PCA plot based on differentially expressed genes (DEGs) from bulk RNA sequencing data from DZ (CD19^+^CD4^−^ GL7^+^Fas^+^CD38^lo^IgD^−^IgG1^+^CXCR4^+^CD86^−^ [sort ii and iii] or CD19^+^CD4^−^ GL7^+^Fas^+^CD38^lo^IgD^−^IgG1^+^CD86^−^ [sort i]) and LZ (CD19^+^CD4^−^ GL7^+^Fas^+^CD38^lo^IgD^−^IgG1^+^CXCR4^−^CD86^+^ [sort ii and iii] or CD19^+^CD4^−^ GL7^+^Fas^+^CD38^lo^IgD^−^IgG1^+^ CD86^+^ [sort i]) IgG1^+^ GC B cells sorted at day 7. Each WT point represents a pool of spleens and dLNs from 4 to 6 mice and each cKO point represents a pool of 7 to 15 mice. Squares are DZ IgG1^+^ GC B cells and circles are LZ IgG1^+^ GC B cells. WT mice are represented by gray and cKO mice are represented by pink. (C) Read counts in counts per million (CPM) of indicated genes in DZ and LZ IgG1^+^ GC B cells. *P < 0.05, **P < 0.01; Student’s *t* test. WT mice are represented by gray circles and cKO mice are represented by pink circles. Each symbol represents pooled cells from 1 sort. (D) GSEA hallmark pathway enrichment analysis in DZ and LZ IgG1^+^ GC B cell samples. Pathways with FDR-controlled *P* values <0.05 were defined as significantly enriched. Pathways represented by pink bars are enriched in cKO mice, gray bars are enriched in WT mice. (E) Volcano plots showing differentially expressed genes, defined as a |log_2_(fold change)| >0.6 and an adjusted *P* value <0.05, between cKO vs WT samples in the DZ and LZ of IgG1^+^ GC B cells. Genes from enriched GSEA hallmark gene sets (indicated in panel B) are colored as indicated. Genes highlighted in purple and black are from a bespoke UPR/ER stress gene list. Genes with a positive fold change are upregulated in cKO mice. NES, normalized enrichment score; ns, not significant.

To further investigate the role of BPTF in GC B cells, we sorted IgG1^+^ GC B cells at day 7 to capture the transcriptional profiles of early GC B cells and performed bulk RNA sequencing (Fig. 2A). Because the DZ:LZ ratio is altered in cKO mice, we sorted and sequenced DZ and LZ IgG1^+^ GC B cells separately. To acquire enough cells, we pooled spleen and dLNs from 4 to 7 WT mice and 7 to 15 cKO mice per sort and sorted all IgG1^+^ GC B cells. Given the severe GC defect in cKO mice, we observed numerous differentially expressed genes between WT and BPTF-deficient DZ (n = 739) and LZ (n = 587) IgG1^+^ GC B cells. PCA revealed a clustering of cKO samples separate from WT samples, demonstrating that BPTF-deficient GC B cells, both DZ and LZ, are transcriptionally distinct from WT GC B cells (Fig. 2B). We particularly noted altered expression of key transcriptional regulators in cKO mice, with a decrease in the expression of *Bcl6* and *Bach2*, which promote GC B cell identity, and a shift toward expression of *Irf4*, *Prdm1*, and *Xbp1*, which promote PC identity (Fig. 2C).^2^^,^^53–55^ Despite BPTF’s established association with MYC activity,^27^^,^^30^^,^^38^^,^^40^^,^^41^*Myc* expression was not significantly different in either the DZ or LZ, nor were GSEA hallmark MYC target pathways differentially expressed in IgG1^+^ GC B cells from WT or cKO mice (Fig. 2C; Fig. S4A).

Within the GSEA hallmark pathway enrichment analysis we found that both DZ and LZ GC B cells from cKO mice upregulated UPR and oxidative phosphorylation (OXPHOS) pathways and downregulated pathways associated with proliferation, including the mitotic spindle, G2M checkpoint, and E2F targets pathways (Fig. 2D; Fig. S4B). Both DZ and LZ IgG1^+^ GC B cells from cKO mice also showed downregulation of the TGF-β signaling pathway, a loss that can lead to LZ GC B cell accumulation.^56^ Additionally, hypoxia and glycolysis pathways were enriched in BPTF-deficient DZ IgG1^+^ GC B cells (Fig. 2D). Somewhat paradoxically, the mTORC1 signaling pathway, which promotes cell growth and proliferation and is essential for GC B cells, was also enriched in BPTF-deficient DZ and LZ GC B cells (Fig. 2D).^10^^,^^11^^,^^57^ While mTORC1 signaling is canonically suppressed in response to cellular stress, previous work has shown that it can be activated in response to ER stress.^58^^,^^59^ Additionally, overactive mTORC1 signaling could itself promote stress by driving unsustainably high levels of OXPHOS and macromolecular synthesis, resulting in ROS accumulation and a buildup of unfolded proteins.^60–62^ We also noted that enrichment of certain pathways within the cKO mice may not indicate increased functional processes, but rather upregulation of individual components of a pathway in response to dysregulation. For instance, investigation of the differentially expressed genes driving enrichment of the mTORC1 signaling pathway in BPTF-deficient GC B cells revealed that some of the genes, including *Nupr1*, *Niban1*, and *Trib3*, are individually prosurvival genes upregulated in response to cellular stress (Fig. 2E).^63–65^ Overall, these data suggest that BPTF-deficient GC B cells may have an altered or dysregulated metabolism, be less proliferative than WT GC B cells, and be under increased cellular stress. The collapse of the IgG1^+^ GC compartment observed between days 7 and 12 in cKO mice suggests that GC B cells may ultimately succumb to BPTF deficiency and either undergo cell death or exit the GC early, as the PC-like shift in transcriptional profiles may indicate.

### BPTF does not promote PC accumulation

**Figure 3:**
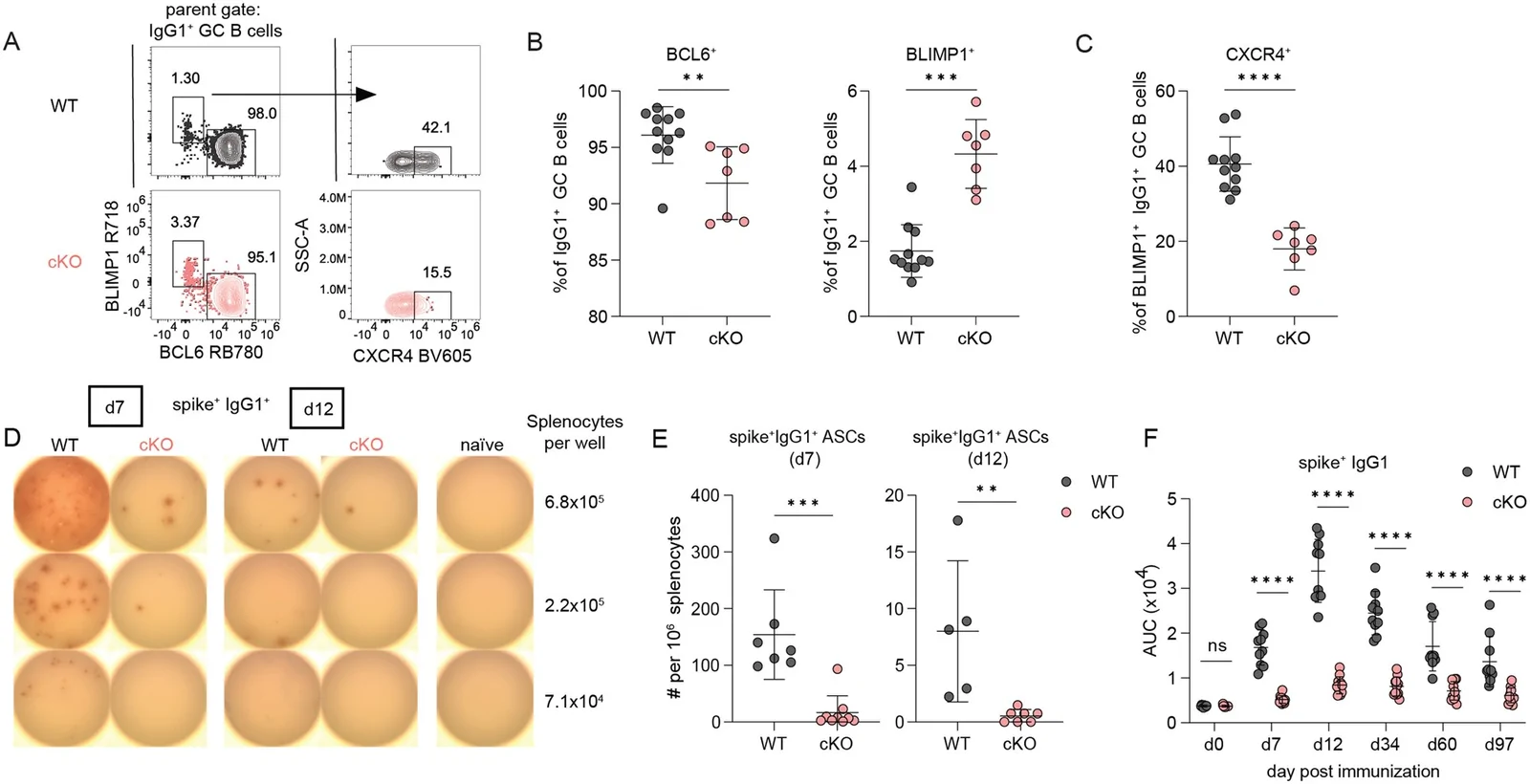
BPTF cKO mice have an impaired antibody response. (A) Representative flow plots of BCL6 and BLIMP1 staining in IgG1^+^ GC B cells and CXCR4 staining in BLIMP1^+^ IgG1^+^ GC B cells in the spleen at day 7 (d7). (B) Frequency of BCL6^+^ or BLIMP1^+^ cells within the IgG1^+^ GC B cell compartment as gated in panel A. Pooled from 2 independent experiments. n = 11 WT and n = 7 cKO mice. (C) Frequency of CXCR4^+^ cells within the BLIMP1^+^ IgG1^+^ GC B cell compartment as gated in panel A. (D) Representative ELISpot wells of d7 and d12 splenocytes identifying S-specific IgG1^+^ ASCs. The number of total splenocytes plated per well is indicated. (E) Quantification of S^+^IgG1^+^ ASCs in the spleen 7 and 12 d postimmunization as identified by ELISpot. n = 5–9 mice per group. d7 and d12 are each representative of 2 independent experiments. (F) Time course of S-specific IgG1 serum antibody titers. Ten or 11 mice per group were bled throughout the course of the experiment at indicated time points. Data are representative of two independent experiments (second experiment time points: d7, d14, d28, d67, d93). **P < 0.01, ***P < 0.001, ****P < 0.0001; Mann-Whitney test. WT mice are represented by gray circles and cKO mice are represented by pink circles. Each symbol represents 1 animal. AUC, area under the curve; ns, not significant; SSC-A, side scatter area.

To corroborate the decrease in *Bcl6* and the increase in *Prdm1* we observed in the RNA sequencing data, we used flow cytometry to investigate the protein levels of BCL6 and BLIMP1 in IgG1^+^ GC B cells (Fig. 3A). At day 7, we observed a lower frequency of BCL6^+^ cells within the BPTF-deficient IgG1^+^ GC B cell compartment (as defined using surface markers: CD19^+^CD4^−^CD138^−^GL7^+^Fas^+^CD38^lo^IgD^lo^) along with an increase in the frequency of BLIMP1^+^ cells (Fig. 3B). However, significantly fewer BLIMP1^+^ IgG1^+^ GC B cells from cKO mice expressed CXCR4, which is essential for proper PC migration to the bone marrow (Fig. 3C).^66^ We next used an ELISpot to quantify the number of S-specific IgG1^+^ ASCs in the spleen to test if GC B cells were exiting the GC early as PCs. At both day 7 (the peak of the PB response) and day 12, we observed that cKO mice had significantly fewer S-specific IgG1^+^ ASCs than WT mice (Fig. 3D, E). Additionally, cKO mice had consistently lower S-specific IgG1 serum antibody titers up to 3 mo postvaccination (Fig. 3F). As the *Ighg1*cre is not GC-specific, we hypothesized that the phenotype at day 7, which we believe to primarily consist of GC-independent PBs, suggests that BPTF may play an additional role at an early B cell stage after antigen stimulation or an independent role in non-GC B cell subsets, even at relatively low expression levels. To test if B cells were capable of differentiation into PCs upon stimulation, we cultured WT B cells with a PC-skewing cocktail of stimulants and a small molecule inhibitor of BPTF (AU1) (Fig. S5A). Similar to what we observed in primary human B cells, mouse B cells treated with AU1 did not expand (Fig. S5B). Additionally, we clearly observed a lower frequency of differentiated PCs in the AU1-treated group compared with the control group as early as day 2 postplating (Fig. S5C, D). These data, together with the rarity of S-specific IgG1^+^ ASCs at day 12 and the low titers at 1, 2, and 3 mo postimmunization, suggest that the skewing BPTF-deficient GC B cells toward a PC-like transcriptional program does not lead to the accumulation of a functional PC pool.

### BPTF is necessary for GC B cell survival

**Figure 4:**
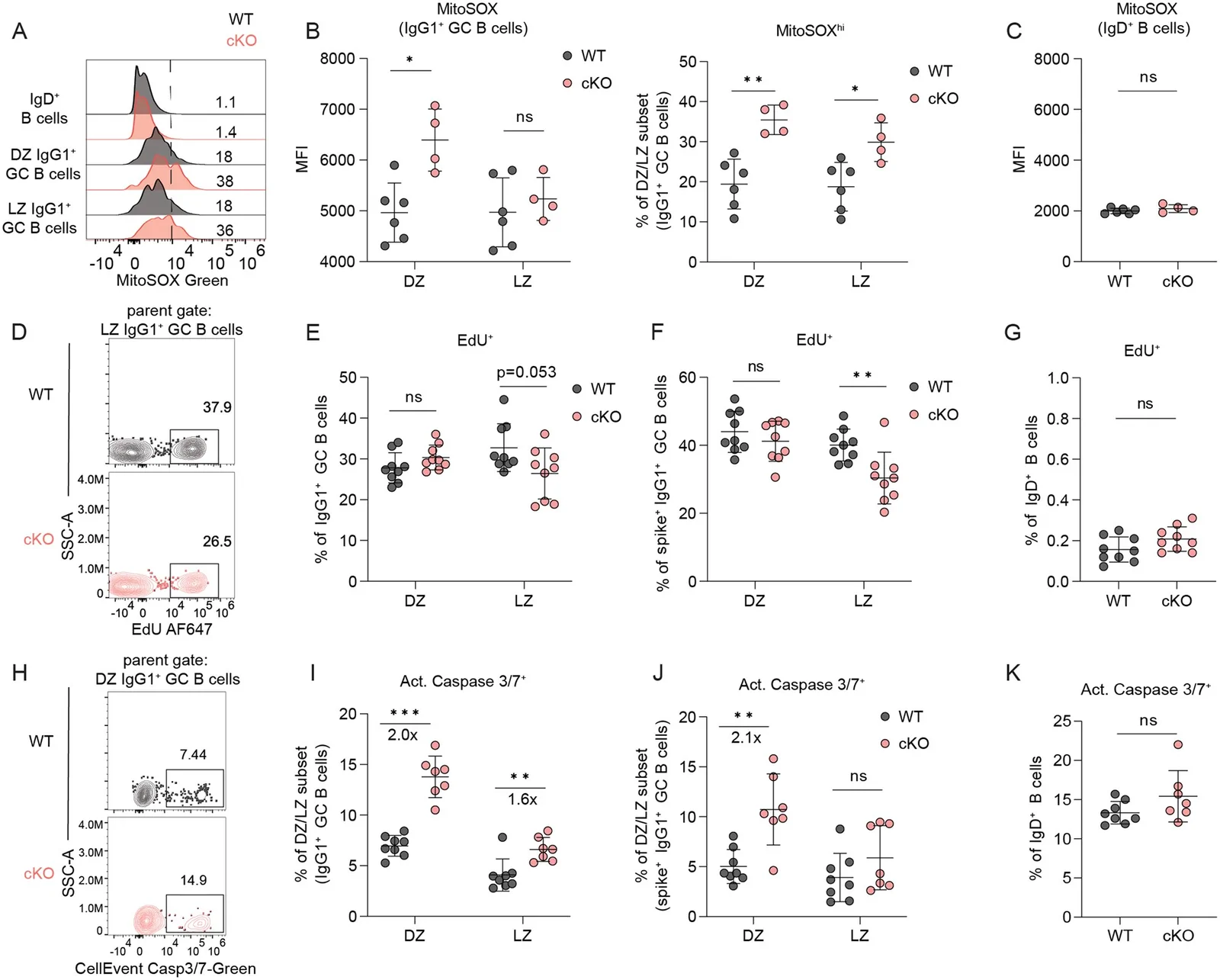
BPTF is necessary for GC B cell survival. (A) Representative flow cytometry histograms showing MitoSOX intensity within IgD^+^ B cells (CD19^+^CD4^−^IgD^+^), DZ IgG1^+^ GC B cells (CD19^+^CD4^−^CD138^−^GL7^+^Fas^+^CD38^lo^IgD^−^IgG1^+^CXCR4^+^CD86^−^), and LZ IgG1^+^ GC B cells (CD19^+^CD4^−^CD138^−^GL7^+^Fas^+^CD38^lo^IgD^−^IgG1^+^CXCR4^−^CD86^+^) of WT and cKO mice. The line indicates MitoSOX^hi^ gate and numbers indicate frequency of each compartment that is MitoSOX^hi^. (B) MitoSOX median fluorescent intensity (MFI) and frequency of MitoSOX^hi^ cells (as gated in panel A) within the DZ and LZ of the IgG1^+^ GC compartments of WT and cKO mice. n = 4–6 mice per group. Data are representative of 2 independent experiments. (C) MitoSOX median fluorescent intensity within the IgD^+^ B cell compartment. n = 4–6 mice per group. Data are representative of 2 independent experiments. (D) Representative flow plots of EdU^+^ cells within the LZ of the IgG1^+^ GC B cell compartment. (E, F) Frequency of EdU^+^ cells within DZ and LZ of total or S-labeled IgG1^+^ GC B cells. n = 9 mice per group. Data pooled from 2 independent experiments.(G) Frequency of EdU^+^ cells within IgD^+^ B cells. n = 9 mice per group. Data pooled from 2 independent experiments.(H) Representative flow plots of activated caspase-3/7 staining within dark zone IgG1^+^ GC B cells. (I, J) Frequency of cells with activated caspase-3/7 within the DZ and LZ of total or S-labeled IgG1^+^ GC B cells. n = 7 or 8 mice per group. Data are representative of 3 independent experiments. (K) Frequency of activated caspase-3/7^+^ cells within the IgD^+^ B cell compartment. n = 7 or 8 mice per group. Data are representative of 3 independent experiments. All data are from spleens at day 7 postimmunization. Data are shown as mean ± SD. *P < 0.05, **P < 0.01, ***P < 0.001; Mann-Whitney test. WT mice are represented by gray circles and cKO mice are represented by pink circles. Each symbol represents 1 animal. ns, not significant; SSC-A, side scatter area.

Given the enrichment of OXPHOS and UPR pathways in BPTF-deficient GC B cells we observed in our GSEA pathway enrichment analysis (Fig. 2D), we hypothesized that these cells would exhibit higher ROS levels, either due to dysregulation or a functional increase in these pathways. While low levels of ROS produced during OXPHOS support cellular homeostasis, prolonged or dysfunctional OXPHOS can lead to an excess of ROS that overwhelms antioxidant defenses. The resulting oxidative stress can ultimately result in DNA and protein damage and an increase in ER stress, which creates a feedback loop that exacerbates oxidative stress within a cell.^67–71^ To quantify mitochondrial-derived ROS levels within BPTF-deficient GC B cells, we stained splenocytes at day 7 postimmunization with MitoSOX. In agreement with our hypothesis, ROS levels were elevated in IgG1^+^ GC B cells from cKO mice (Fig. 4A, B), but not in bystander IgD^+^ B cells (Fig. 4C).

ROS accumulation can have detrimental effects on a cell, including DNA damage, which can negatively impact the cell’s proliferative ability.^68^^,^^72^ This observation, along with the GSEA pathway analysis, led us to next test the proliferative capacity of GC B cells in WT and cKO mice. We immunized WT and cKO mice and administered EdU 1 h before harvest on day 7. We observed a significant decrease in the frequency of LZ cells in S phase in both the total and S-labeled IgG1^+^ GC B cell compartments of cKO mice (Fig. 4D–F). However, despite enrichment of GSEA hallmark pathways associated with proliferation in both the DZ and LZ IgG1^+^ GC B cells of WT mice, frequencies of DZ IgG1^+^ GC B cells in S phase in WT and cKO mice were similar (Fig. 4E, F). We did not observe a significant difference in the relatively small population of IgD^+^ B cells that had taken up EdU (Fig. 4G). The decrease in the frequency of LZ IgG1^+^ GC B cells in S phase in cKO mice, together with the increased ratio of LZ:DZ IgG1^+^ GC B cells we observed (Fig. 1I), suggests that BPTF may be required for LZ GC B cells to re-enter the cell cycle and transition back to the DZ. However, this defect did not translate to fewer DZ GC B cells in S phase, despite the loss of proliferation-associated pathways in our GSEA pathway analysis (Fig. 2D). We attempted to compare the frequency of DZ IgG1^+^ GC B cells in G2/M phase between WT and cKO mice using EdU/Hoechst dual staining but could not demarcate DZ and LZ IgG1^+^ GC B cells. Thus, we were unable to determine whether DZ IgG1^+^ GC B cells had a functional defect in post–S phase proliferation using this method.

However, a potential effect of DZ GC B cells undergoing late cell-cycle arrest is increased cell death, either via direct mechanisms mediated by BPTF or via indirect responses to cellular stress. To investigate this possibility, we stained splenocytes with an active caspase-3/7 marker 7 d postimmunization. We observed an approximate 2-fold increase in the frequency of BPTF-deficient DZ IgG1^+^ GC B cells that were positive for activated caspase-3/7 and a 1.5-fold increase in the LZ of cKO mice (Fig. 4H, I). While we saw this effect mirrored in the S-labeled DZ IgG1^+^ GC B cells, we did not observe a significant difference in caspase activation in S-specific LZ IgG1^+^ GC B cells (Fig. 4J). Despite increased GC B cell death in cKO mice, we observed no difference in caspase-3/7 activation in IgD^+^ B cells at day 7 (Fig. 4K). Whether due to cell cycle arrest or other ramifications of *Bptf* loss, we can conclude that a contributing factor to the impaired GC B cell response that we observed in our cKO mice is cell death.

## Discussion

As a key component of a chromatin remodeling complex, BPTF can regulate multiple facets of cellular function and survival, both directly and indirectly. Other chromatin remodelers have been implicated in the function and maintenance of GC B cells, although the mechanisms vary.^14–18^ For instance, B cell–specific deletion of the shared SRG3 subunit of SWI/SNF family members leads to a loss of BCL6-mediated BLIMP1 repression, resulting in fewer GC B cells but more short-lived PCs.^15^ Deletion of other subunits within only some SWI/SNF family member complexes led to different GC B cell phenotypes, including early GC exit as immature MBCs, GC B cell loss following upregulation of inflammatory signatures, and downregulation of cell cycle pathways.^16–18^ Additionally, SMARCA5, an ATPase utilized by the ISWI family of chromatin remodelers, to which the NURF complex belongs, is necessary for proliferation and GC B cell programming in pre-GC activated B cells.^14^ While BPTF can interact with SMARCA5, its canonical complex (NURF) utilizes the other ATPase associated with ISWI chromatin remodelers, SMARCA1.^73^

Due to the number of studies that have described BPTF as a driver of proliferation^20–22^^,^^27^^,^^29–35^^,^^37^^,^^38^ and the decreased ratio of DZ:LZ IgG1^+^ GC B cells we observed in cKO mice, we were surprised that the frequency of DZ GC B cells in S phase was comparable between WT and cKO mice. However, this does not necessarily mean that BPTF-deficient DZ GC B cells can successfully complete the cell cycle. Therefore, further studies are needed to address the role of BPTF in the proliferation of GC and non-GC activated B cells.

Interestingly, the LZ GC B cell population from cKO mice contained a reduced frequency of cells in the S phase of the cell cycle, a transition associated with MYC activity.^7^^,^^9^ Several studies have reported that BPTF is necessary for MYC transcriptional activity.^27^^,^^30^^,^^38^^,^^40^^,^^41^ Therefore, as MYC is essential for GC B cell function, we hypothesized that MYC target pathways would be downregulated in BPTF-deficient GC B cells.^7–9^ However, we observed no differences in MYC target pathways between the DZ or LZ of cKO and WT mice. Because *Myc* is expressed at low levels in only a small subset of GC B cells at any given time, it is possible that we were not able to capture the impact of BPTF loss on MYC target pathways in the GSEA analysis, although our independent analyses of DZ and LZ GC B cells may minimize this concern.^7^^,^^9^

Although the relationship between BPTF and proliferation in the GC was somewhat difficult to interpret, we were able to capture shifts in identity-defining transcriptional programming within the GC. We observed that BPTF loss led to expression of genes associated with PC fate, including *Prdm1*, *Irf4*, and *Xbp1*, within cells exhibiting a GC B cell phenotype. This phenomenon has been previously reported and, similar to what we observed in our model, was associated with a reduction of both GC B cells and PCs.^74^

In association with this transcriptional shift in GC B cells, we observed increased expression of cellular stress genes and an accumulation of ROS, suggesting that these cells may not be functioning optimally. While further studies are needed to determine which of these observations are a direct consequence of BPTF loss and which are part of an apparent stress-response cascade, the ultimate consequence of BPTF loss in GC B cells appears to be cell death. Finally, potentially owing to the loss of GC B cells, cKO mice had diminished antigen-specific MBC and PC populations at a memory time point.

Overall, we show that loss of BPTF in GC B cells results in an impaired GC response and fewer antigen-specific MBCs and PCs following immunization. Based on transcriptional profiling, we propose that, in the absence of BPTF, GC B cells upregulate prosurvival genes in response to increased cellular stress, either inherent to GC B cells or uniquely generated by BPTF loss. Despite cells’ attempts at stress rescue, we found that BPTF-deficient GC B cells were more prone to cell death, thereby impairing vaccine-induced humoral immune responses. However, determining the initial epigenetic and transcriptional changes a GC B cell undergoes upon BPTF deletion requires additional studies. Characterization of BPTF-DNA interactions and BPTF-driven shifts in chromatin architecture will help pinpoint direct changes in gene availability that could inform the precise mechanisms by which BPTF regulates B cell fate and survival.

## Supplementary Material

vkag126_Supplementary_Data

## Data Availability

Additional data supporting this manuscript is publicly available at WashU Medicine’s Digital Commons Data@Becker Repository via https://doi.org/10.17632/x8xyxc8r6v. Raw sequencing data have been deposited at Sequence Read Archive under BioProject PRJNA1450725. The mouse bulk RNA sequencing count matrix has been deposited at Gene Expression Omnibus under GSE327666. Processed human single-cell RNA sequencing data have been deposited on Zenodo at https://doi.org/10.5281/zenodo.19500486.
