# Supplementary material for: BPTF is essential for vaccine-induced germinal center B cell responses
Source: J Immunol. 2026 Jun 20;215(6):vkag126. doi: 10.1093/jimmun/vkag126 (PMC13282703; doi:10.1093/jimmun/vkag126)
Supplement: vkag126_Supplementary_Data [file vkag126_supplementary_data.pdf]

# Supplementary material:

# BPTF is essential for vaccine-induced germinal center B cell responses

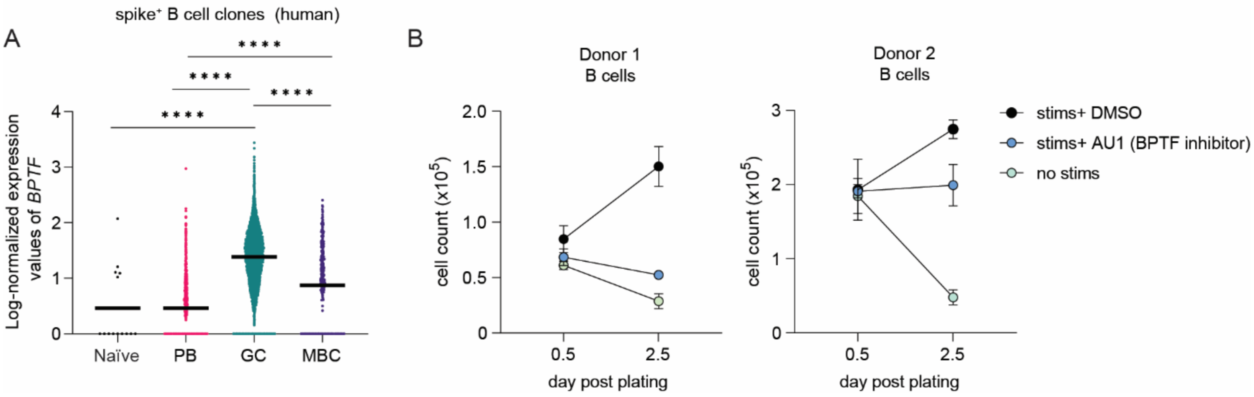


**Supplemental Figure 1. BPTF is highly expressed in GC B cells.** (A) *BPTF* expression in spike specific B cell populations in participants after receiving the Pfizer-BioNtech SARS-CoV-2 mRNA-LNP vaccine (BNT162b2) or the Moderna SARS-CoV-2 mRNA-LNP vaccine (mRNA-1273). Paired single cell gene expression and BCR RNA-sequencing were performed on cells collected from blood and draining lymph node of participants. Spike-specific clones were identified via a monoclonal antibody screening ELISA. PB: plasmablasts, GC: Germinal center B cells, MBC: memory B cells. Each individual dot represents a single cell; n= 14 (naïve), 1838 (PBs), 28313 (GC B cells), 357 (MBCs). All cells were compiled from early and late time points (1-29 weeks post-booster). Line represents median. Multiplicity-adjusted Student's t-test. *p<0.05, ** p<0.01, ***p<0.001, ****p<0.0001.(B) Number of live B cells ($CD19^{+}CD14^{-}$ $CD3^{-}$) recovered from *in vitro* culture at the indicated time points. Primary B cells enriched from healthy human blood were treated with DMSO or a small molecule inhibitor of BPTF (AU1) along with the stimulant cocktail (CD40L, R848, and a-IgG/M/A) at hour 0. Each point represents the mean ± SD of 3 technical replicates. Donor 1 and donor 2 experiments were performed independently.

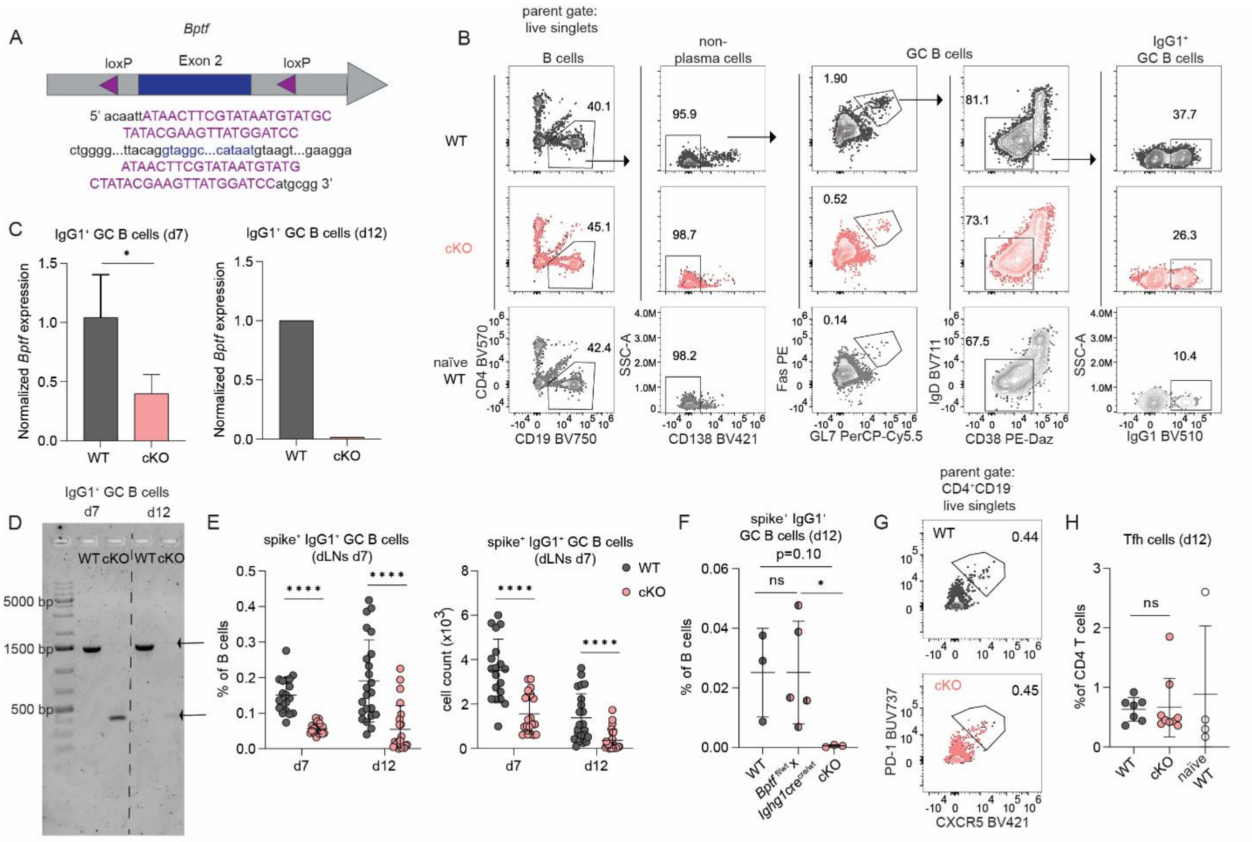


**Supplemental Figure 2. Validation of cKO mice.** (A) Bptf floxed mouse design. LoxP sites were inserted flanking exon 2 within the intron regions as indicated by sequence. (B) Flow gating strategy for IgG1$^+$ GC B cells. Representative of day 7 spleen. (C) qPCR analysis of *Bptf* expression in sorted IgG1$^+$GC B cells (CD19$^+$CD4$^-$ GL7$^+$Fas$^+$CD38$^{lo}$IgD$^-$IgG1$^+$) from the spleen. Day 7: n=4 mice per group. Day 12: spleens were pooled from n=4 WT mice and n=6 cKO mice into n=1 WT and n=1 cKO samples. Data from each time point represents 1 experiment. (D) Representative DNA gel confirming the excision of exon 2 in sorted IgG1$^+$ GC B cells from the spleen at days 7 and 12 post-immunization. Day 7: n=4 mice (1 representative shown here). Day 12: spleens were pooled from n=4 WT mice and n=6 cKO mice into n=1 WT and n=1 cKO samples. Day 12 IgG1$^+$GC lane was overloaded. PCR primers flank the loxP sites inserted on either side of *Bptf* exon 2 and, within cKO mice, results in a wild-type product of 1543 base pairs

and a deletion product of 377 base- both indicated by arrows. Data representative of 1 experiment. (E) Frequency and number of spike$^{+}$IgG1$^{+}$ GC B cells (CD19$^{+}$CD4$^{-}$CD138$^{-}$GL7$^{+}$Fas$^{+}$CD38$^{lo}$IgD$^{-}$IgG1$^{+}$spike$^{+}$) in draining lymph nodes 7- and 12- days after immunization measured by flow cytometry. Day 7 pooled from 3 independent experiments and day 12 pooled from 4 independent experiments. n=19-23 mice per group. Each point represents pooled ipsilateral inguinal + iliac draining lymph nodes from one animal. (F) Frequency of spike$^{+}$IgG1$^{+}$ GC B cells (CD19$^{+}$CD4$^{-}$CD138$^{-}$GL7$^{+}$Fas$^{+}$CD38$^{lo}$IgD$^{-}$IgG1$^{+}$spike$^{+}$) in spleens of *Bptf*$^{fl/wt\ or\ fl/fl\ or\ wt/wt}$ x *Ighg1*cre$^{cre/wt}$ mice 12 days post-immunization, measured by flow cytometry. Data representative of 1 experiment. (G) Representative flow plots of T follicular helper cell staining (CD4$^{+}$CD19$^{-}$CXCR5$^{+}$PD-1$^{+}$) in the spleen 12-days post-immunization. (H) Frequency of Tfh cells in the spleen 12 days post immunization as gated in (G). n=7-9 mice per immunized group and n=4 naïve mice. Immunized data was pooled from two independent experiments. Naïve mice were pooled from 4 independent experiments. Data shown as mean ± SD. *p<0.05, ** p<0.01, ***p<0.001, ****p<0.0001. Mann-Whitney test. WT mice are represented by gray circles and cKO mice are represented by pink circles. Each symbol represents one animal.

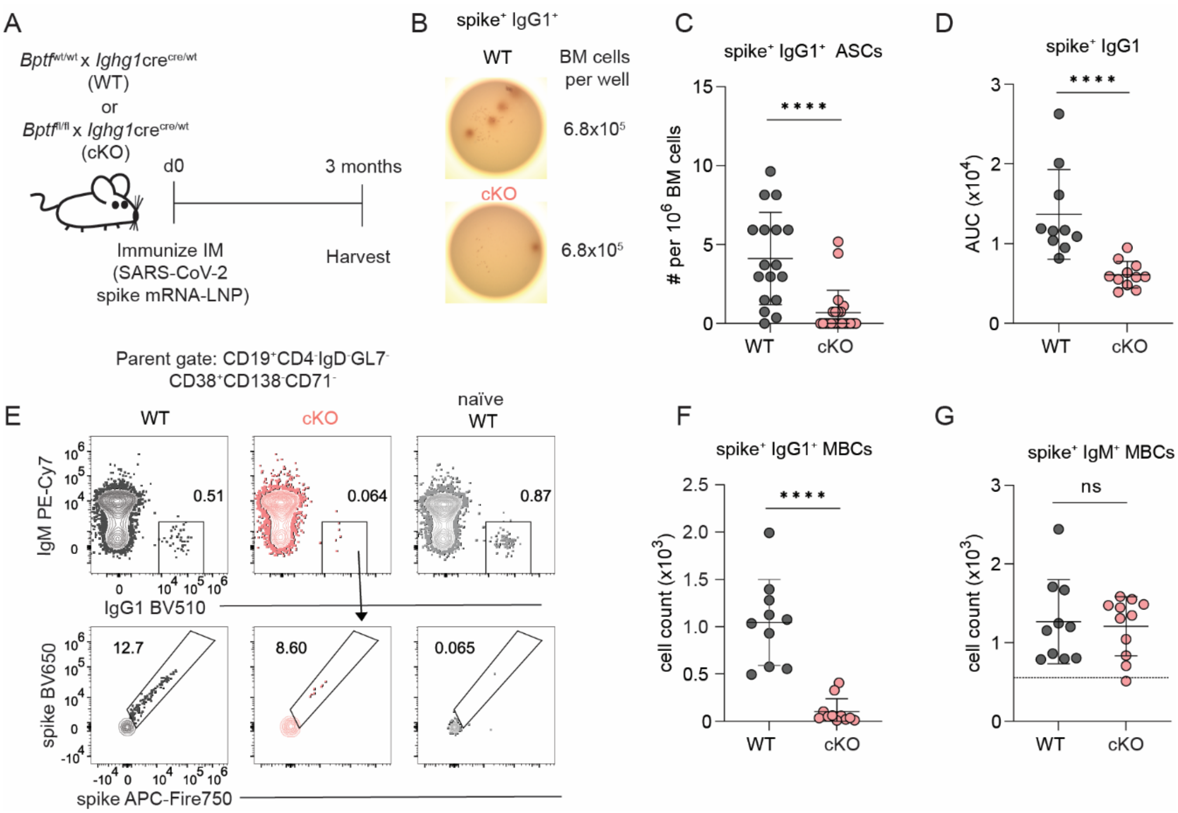


**Supplemental Figure 3. BPTF cKO mice have fewer antigen-specific MBCs and PCs at a memory time point.** (A) Experimental design. *Bptf*$^{\text{wt/wt or fl/fl}}$x*Ighg1*cre$^{\text{cre/wt}}$ mice were immunized intramuscularly with a single dose of 10 µg of SARS-CoV-2 spike mRNA-LNP vaccine. Spleen, bone marrow, and serum were harvested 3 months post-vaccination. (B) Representative ELISpot wells of bone marrow 3 months post-immunization identifying spike-specific IgG1$^{+}$ antibody secreting cells. The number of cells plated per well is indicated. (C) Number of spike$^{+}$IgG1$^{+}$ ASCs in bone marrow 3 months post-immunization as quantified by ELISpot. n=17 WT and n= 22 cKO mice. Pooled from two independent experiments. (D) Serum antibody titers of spike$^{+}$IgG1 at 3 months post-immunization. n=10 WT and n= 11 cKO mice. Data is representative of two independent experiments. (E) Representative flow plots identifying spike$^{+}$IgG1$^{+}$ MBCs (CD19$^{+}$CD4$^{-}$IgD$^{-}$GL7$^{-}$CD38$^{+}$CD138$^{-}$CD71$^{-}$IgM$^{-}$IgG1$^{+}$spike$^{+}$) in the spleen 3 months post-immunization. (F) Number of spike$^{+}$IgG1$^{+}$ MBCs in the spleen at 3 months post-

immunization as gated in (E). n=10 WT and n= 11 cKO mice. Data is representative of two independent experiments. (G) Number of spike$^{+}$IgM$^{+}$ MBCs (CD19$^{+}$CD4$^{-}$IgD$^{-}$GL7$^{-}$CD38$^{+}$CD138$^{-}$CD71$^{-}$IgM$^{+}$IgG1$^{-}$spike$^{+}$) within the spleens of WT and cKO mice at 3 months post-immunization. Dotted line indicates background spike staining on IgM$^{+}$ MBCs in a naïve mouse. Representative of 2 independent experiments. Data shown as mean ± SD. ****p<0.0001. Mann-Whitney test. WT mice are represented by gray circles and cKO mice are represented by pink circles. Each symbol represents one animal.

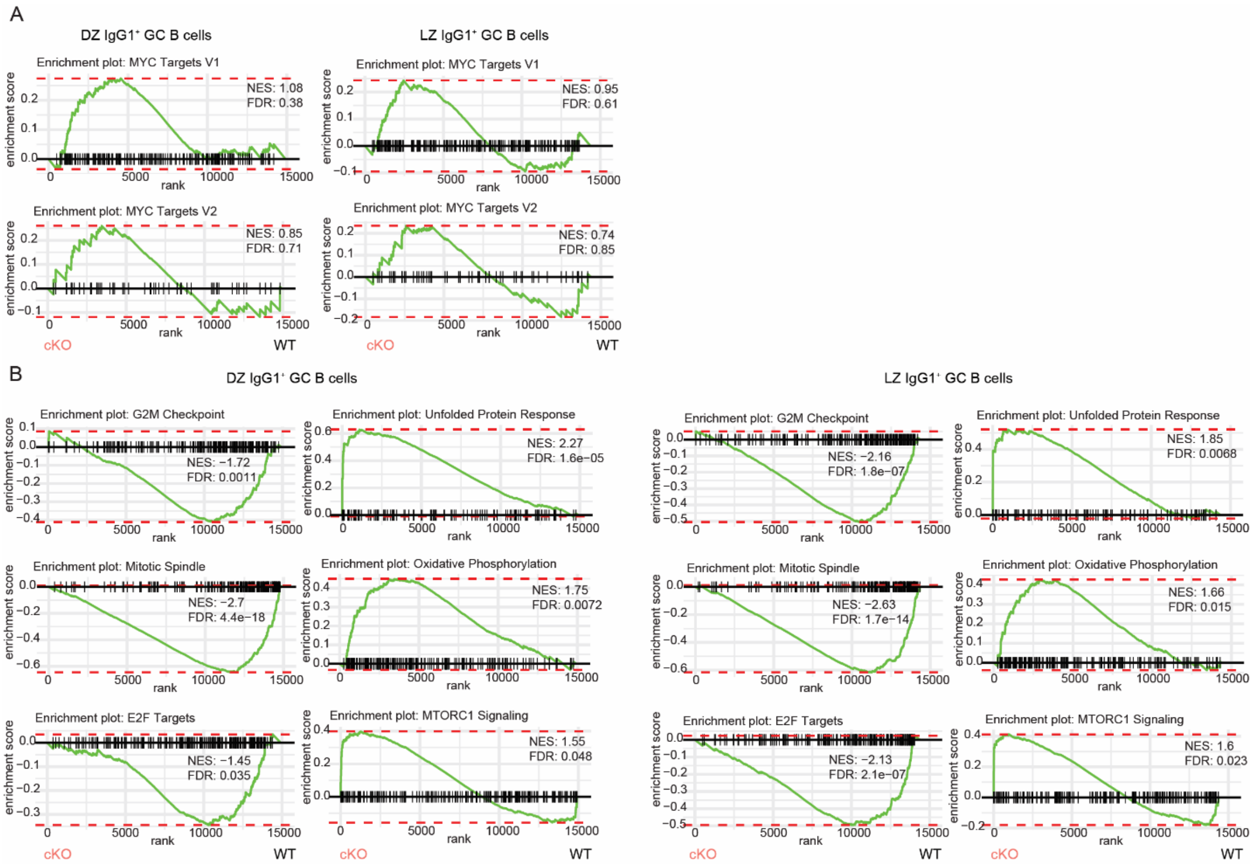


**Supplemental Figure 4. GSEA enrichment plots for key hallmark pathways.** (A) GSEA enrichment plots for MYC target pathways or (B) key GSEA hallmark pathways from Figure 2D in dark zone and light zone IgG1$^+$ GC B cells. Peaks with a negative enrichment score are enriched in WT samples and peaks with a positive enrichment score are enriched in cKO samples.

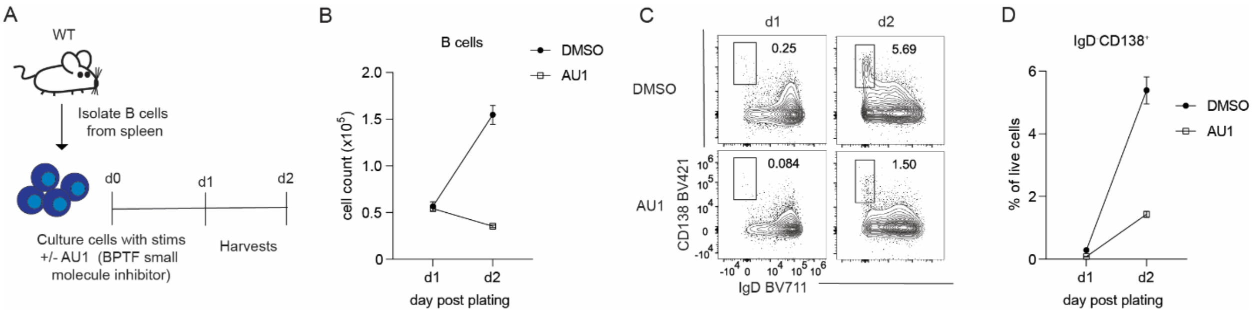


**Supplemental Figure 5. BPTF inhibition impairs formation of a PC population.** (A) Experimental design. Naïve B cells were enriched from the spleen of a WT mouse and plated with DMSO or a small molecule inhibitor of BPTF (AU1) along with the stimulant cocktail (CD40L, IL-4, and R848) at hour 0. Samples were harvested 1 or 2 days after plating and analyzed by flow. (B) Number of live B cells ($CD19^+$) recovered from culture at indicated time points. (C) Representative flow plots of $CD138^+IgD^-$ cells in DMSO and AU1-treated conditions at day 1 and day 2. (D) Quantification of $CD138^+IgD^-$ cells as gated in (C). Data from 1 experiment. Each point represents the mean ± SD of 2-3 technical replicates. Black circles represent the DMSO treatment, white squares represent the AU1 treatment.
